# A novel splice variant in *EMC1* is associated with cerebellar atrophy, visual impairment, psychomotor retardation with epilepsy

**DOI:** 10.1002/mgg3.352

**Published:** 2017-12-22

**Authors:** Thenral S. Geetha, Lokesh Lingappa, Abhishek Ravindra Jain, Hridya Govindan, Nitin Mandloi, Sakthivel Murugan, Ravi Gupta, Ramprasad Vedam

**Affiliations:** ^1^ Medgenome Labs Bommasandra Bangalore India; ^2^ Rainbow Hospital Hyderabad India

**Keywords:** clinical exome, developmental delay, *EMC1*, epilepsy, Indian population, psychomotor retardation, South Asian, splice variant

## Abstract

**Background:**

Several genes have been implicated in a highly variable presentation of developmental delay with psychomotor retardation. Mutations in *EMC1* gene have recently been reported. Herein, we describe a proband born of a consanguineous marriage, who presented with early infantile onset epilepsy, scaphocephaly, developmental delay, central hypotonia, muscle wasting, and severe cerebellar and brainstem atrophy.

**Methods:**

Genetic testing in the proband was performed using custom clinical exome and targeted next‐generation sequencing. This was followed by segregation analysis of the variant in the parents by Sanger sequencing and evaluation of the splice variant by RNA sequencing.

**Results:**

Clinical exome sequencing identified a novel homozygous intronic splice variant in the *EMC1* gene (chr1:19564510C>T, c.1212 + 1G>A, NM_015047.2). Neither population databases (ExAC and 1000 genomes) nor our internal database (*n* = 1,500) had reported this rare variant, predicted to affect the splicing. RNA sequencing data from the proband confirmed aberrant splicing with intron 11 retention, thereby introducing a stop codon in the resultant mRNA. This nonsense mutation is predicted to result in the premature termination of protein synthesis leading to loss of function of the EMC1 protein.

**Conclusion:**

We report, for the first time the role of aberrant *EMC1* RNA splicing as a potential cause of disease pathogenesis. The severe epilepsy observed in our study expands the disease‐associated phenotype and also emphasizes the need for comprehensive screening of intronic splice mutations.

## 1 INTRODUCTION

*EMC1* (MIM616846) is subunit 1 of the endoplasmic reticulum membrane protein complex (EMC), an evolutionarily conserved complex involved in the process of ER‐associated degradation (ERAD). The lumen of the endoplasmic reticulum (ER) is the major site for proper protein folding, post‐translational modification, assembly of multimeric structures, and transport of these proteins to other organelles and cells through the secretory pathway. Misfolded and mutant proteins are retained in the endoplasmic reticulum and degraded by ERAD, an ubiquitin and proteasome‐dependent process. Perturbations in the function and integrity of the ER or changes in calcium homeostasis due to mutations in ER‐related genes result in the pathogenesis of diseases including epilepsy (Kang & Macdonald, 2009), neurodegenerative disease (Xiang, Wang, Zhang, & Han, 2017), metabolic diseases (Wang, Song, Brancati, & Segatori, 2011), Pelizaeus–Merzbacher, Krabbe leucodystrophies, vanishing white matter disease, and Charcot–Marie–Tooth neuropathies (Janer et al., 2016; Volpi, Touvier, & D'Antonio, 2017). The physiological roles of the *ERAD* pathway and *EMC1* remain largely unknown; however, several recent studies of animal models and human diseases have shed light on *EMC1* function.

Mutations in the *EMC1* gene (MIM616846), an integral part of the ER membrane complex, have been recently implicated in individuals affected with cerebellar atrophy, visual impairment, and psychomotor retardation (MIM616875). In this study, we report a novel splice variant in the *EMC1* gene in a proband born of a consanguineous marriage, who presented with early infantile onset epilepsy, muscle wasting, severe psychomotor retardation, and developmental delay.

## 2 MATERIALS AND METHODS

### 2.1 Ethical compliance

The study was approved by the Rainbow Hospital ethics committee. Informed consent was obtained, and clinical evaluation including radiological profiling was done. Genomic DNA was extracted from blood samples collected from the proband (at 4 years and 10 months of age) and his parents using the manufacturer's protocol (Qiagen).

### 2.2 Clinical exome sequencing and bioinformatics analysis

An in‐house custom clinical exome panel covering ~6,400 genes implicated in various Mendelian disorders was used. The library was enriched using a custom Nimblegen panel and sequenced as 2 × 100 bp paired‐end reads on an Illumina Hiseq2000 (Illumina, CA) machine according to the manufacturer's protocol. After obtaining the data, we used GATK best practices variant‐calling pipeline to identify germline variants. Briefly, the adapters from the raw reads were trimmed using fastq‐mcf; trimmed reads were mapped on the human reference genome (hg19) using BWA (Li & Durbin, 2010); duplicate reads were removed using Picard and then realignment, recalibration, and variant calling were performed using GATK (McKenna et al., 2010). The identified variants were annotated to genes using the VEP program against the Ensembl release 84 human gene model. Deep annotation of the germline variants was performed using an in‐house VariMAT pipeline whereby the annotation against licensed latest HGMD, ClinVar, SwissVar, population databases (1000Genome Phase 3, ExAC, NHLBI, dbSNP, 1,000, Japanese and our internal Indian specific database) was used. Further, we annotated the variant against *in silico* prediction algorithms (PolyPhen‐2, SIFT, Mutation Taster2, and LRT). The variants were analyzed using Varminer (in‐house variant interpretation tool), and the pathogenicity of the variants were assessed based on 2015 ACMG guidelines.

### 2.3 Sanger sequencing

Sanger sequencing was performed for the prioritized *EMC1* gene (NG_032948.1) variant, following PCR (polymerase chain reaction) amplification using pxlence primers (Cat no: PXL‐A0003493) on an ABI 3730 genetic analyzer (Applied Biosystems, CA).

### 2.4 RNA sequencing and bioinformatics analysis

RNA was isolated from blood by the conventional Trizol (Sigma) method. The mRNA is selected by a Poly(A) mRNA magnetic isolation module (Dynabeads), and processed using RNA library preparation according to the manufacturer's protocol (NEB). The RNA was sheared (50–150 bp) and cDNA generated using random priming during first and second strand synthesis. The double‐stranded cDNA fragments were ligated with adapters and sequenced on Hiseq2500. The reads were preprocessed to remove adapters and low‐quality bases using cutadapt and fastq‐mcf programs. The preprocessed reads were aligned to the human genome (hg19) and transcriptome (Ensembl Gene Model) using the STAR aligner program.

## 3 RESULTS

### 3.1 Clinical synopsis

**Figure 1:**
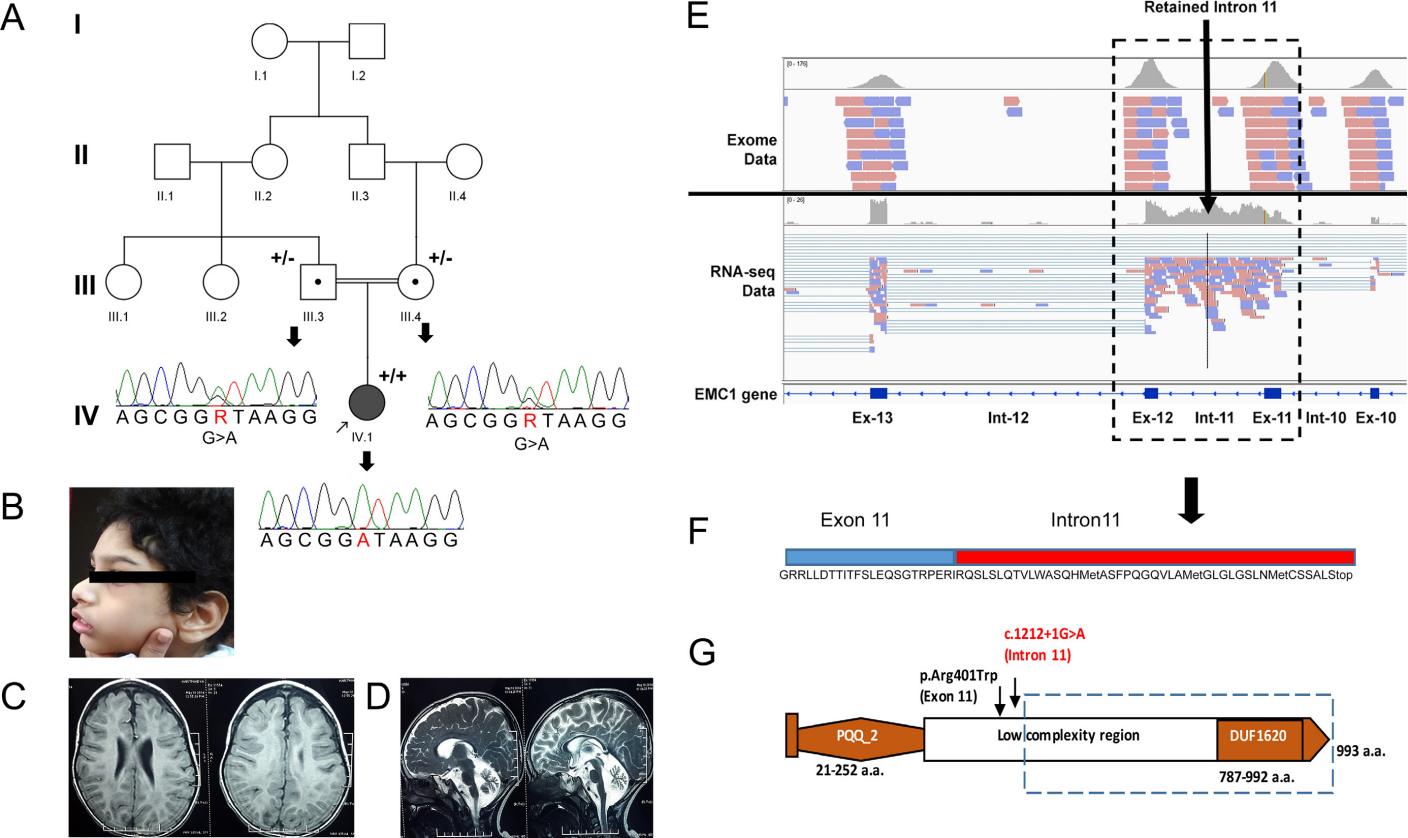
(A) Schematic representation of the consanguineous partial pedigree of the family: circles indicate female and squares indicate male, filled‐in squares indicate affected; circles/squares with dots are obligate carriers; arrow indicates the consultand. The Sanger sequencing chromatogram of the splice variant (c.1212 + 1G>A; NM_015047.2; NG_032948.1) detected in the parents (III‐5, III‐6) and proband (IV‐1) are represented as “+” for variant/mutant allele and “−” for wild‐type allele. The nucleotide numbering reflects cDNA numbering; the initiation codon is codon 1 according to the journal guidelines (http://www.hgvs.org/mutnomen); (B) Side profile photograph of the affected proband showing scaphocephaly; (C, D) MRI Brain Sections of the proband (C) T1 axial view showing supratentorial cerebral atrophy (D) T2 sagittal view showing cerebellar and brainstem atrophy; (E) Schematic representation from Integrative Genomics Viewer showing exome and RNA sequencing data reads encompassing exon 11 and exon 12. The RNA reads show intron 11 retention in the aligned data (F) This peptide sequence represents partial exon 11 (in blue) followed by retained intron 11 sequence (in red); translated upstream (frame 1) till the first stop codon; (G) Schematic representation of the domains of *EMC1*, where PQQ_2 represents the quinoprotein alcohol dehydrogenase‐like domain and DUF1620 represents an uncharacterized domain of unknown function 1,620 with the two variants identified in this study (p.Arg401Trp and c.1212 + 1G>A; NM_015047.2; NG_032948.1). The dotted line indicates protein domains which may be lost due to the mutation detected

The proband (Figure 1b) is the first born of a consanguineous marriage (parents are first cousins) with uneventful antenatal and perinatal history. He initially presented at 18 months of age with global developmental delay and seizures in the form of myoclonic jerks and generalized tonic–clonic seizures (GTCS). Seizures were first noted at 5 months with the frequency increasing from once a month to 1–2/week by 18 months. The predominant seizure type was GTCS and occasional myoclonic jerks. By 4.5 years, the frequency of seizures increased to 3–4/day, primarily GTCS in nature. There was no status epilepticus documented. There was one incident of fever‐triggered seizure with transient worsening of the symptoms. Multiple antiepileptic medications were tried including Sodium valproate, Levetiracetam, Clobazam, Lacosamide, Zonisamide, all with poor response.

At 18 months of age, the proband's head circumference was 43 cm (<3rd percentile) with scaphocephaly and deep‐set eyes. The fundi were normal and tracking light. He showed poor swallowing coordination, hypoplastic nipples, central hypotonia, and with sluggish reflexes and antigravity movements present in all four limbs. Left upper limb dystonia was noted during this examination. The child had scoliosis and severe disuse atrophy of muscles. He had severe delayed milestones. On examination at 4 years 10 months, he had partial head control and roll over. He was able to recognize his parents' voice but was unable to speak and had no eye contact, indicating cerebral visual impairment. He grasped objects when placed in his hands.

Results of standard newborn screening test for enzymes for inherited metabolic disorders and nerve conduction studies were within normal limits. His electroencephalogram (EEG) was suggestive of liability of occipital onset seizure disorder (right > left) with diffuse cerebral dysfunction. Ophthalmic evaluation showed cortical visual impairment and optic disc pallor. Magnetic resonance imaging (MRI) of brain demonstrated severe cerebellar atrophy without signal changes and brainstem atrophy. Myelination was age appropriate with mild diffuse atrophy in the supratentorial compartment (Figure 1c,d). The clinical differential diagnoses included (1) infantile neuronal ceroid lipofuscinosis (NCL), (2) mitochondriopathy, and (3) congenital disorders of glycosylation. However, none of the three differential diagnoses could entirely explain the MRI manifestations and symptoms observed in the child. The child did not have variable systemic manifestations generally observed in congenital disorders of glycosylation. Neither did he have episodic worsening (such as febrile illness), commonly associated with acute metabolic stress, in mitochondrial disorders. To obtain a diagnosis, the proband was sequenced for the clinical exome panel.

### 3.2 Identification of the splice variant in *EMC1* gene

On sequencing the proband, a total of 5 Gb of data were generated, of which 90.14% of the bases had >= Q30 quality and more than 99% of the reads aligned to the human genome. A total of 15,021 on‐target variants with at least 10× depth of coverage were detected. Variants with >5% minor allele frequency in various population‐based databases (ExAC, 1000Genome, NHLBI, dbSNP, 1000Japanese, Internal database) were excluded. The remaining variants were sequentially ranked and filtered based on reported variants in disease databases, evolutionary conservation, in silico prediction, and clinical relevance with symptoms presented in the proband. Homozygous and compound heterozygous variants were prioritized, based on the assumed recessive mode of inheritance of the disease in this family.

Following the prioritization, we identified a homozygous variant in intron 11, at the 5′ donor splice site affecting the G residue at position +1, of the *EMC1* gene (chr1:19564510C>T, c.1212 + 1G>A, NM_015047.2). This splice site is highly conserved across species with a significant CADD score (raw score: 5.7 and phred: 26.8; v1.3) and was not detected in any of the public databases (1,000 genomes, ExAC, NHLBI, dbSNP, 1,000 Japanese, Internal database). This variant was predicted to alter the splice site leading to aberrant splicing by in silico tools Human splicing finder and MutationTaster2 (Desmet et al., 2009; Schwarz, Cooper, Schuelke, & Seelow, 2014). Additionally, a rare missense variant affecting codon 401 in exon 11 (chr1:19564522; G>A, p.Arg401Trp), resulting in the substitution of Tryptophan for Arginine was also detected. The Arginine residue is highly conserved across mammals, with a minor allele frequency of <0.01% in the ExAC database. Recently, loss–of‐function mutations in *EMC1* were detected in three families with cerebral and cerebellar atrophy (Harel et al., 2016). Both variants were in a ~1.5 Mb homozygous stretch (chr1:19476307‐21016838). Segregation of the splice site homozygous variant was confirmed by Sanger sequencing in the parental samples (Figure 1a). There was no intermarriage among direct descendants of the great‐grandparents other than of the proband's parents, making him the only possible subject autozygous for the rare mutation in either great grandparent.

### 3.3 Confirmation of aberrant splicing by RNA sequencing

The phenotypic effect of the splice mutation on mRNA splicing was assessed by transcriptome sequencing. As the gene is ubiquitously expressed, the effect on splicing was assessed in RNA derived from blood cells. More than 33 million reads were generated and ~95% were mapped using HISAT. Analysis of the *EMC1* gene transcripts revealed aberrant splicing with intron 11 retention during transcription (Figure 1e). Translation of the resultant mRNA is predicted to result in the insertion of 42 new amino acids following exon 11 (codon 404; NM_015047.2), and premature truncation of the protein due to the stop codon (Figure 1f), resulting in the loss of a large part of the EMC1 protein, including the DUF1620 domain encompassing exons 12–23 (Figure 1g).

## 4 DISCUSSION

The *EMC1* in humans is a highly conserved gene on chromosome 1, coding for a 993 amino acid transmembrane protein, which is a component of the ER membrane protein complex (EMC). Based on the literature available, EMCs have been suggested to be involved in ubiquitin recognition and protein dislocation in mammals (Christianson et al., 2011); they have been shown in yeast to act as chaperones in protein folding, facilitators of phospholipid transfer from ER to mitochondria (Lahiri et al., 2014). Loss of function of genes in ER complex has been shown to cause accumulation of substrate‐specific misfolded proteins (Jonikas et al., 2009). The human EMC1 protein consists of two conserved domains, a quinoprotein alcohol dehydrogenase‐like domain (PQQ_2) spanning residues 28–242, and an uncharacterized domain of unknown function 1620 (DUF1620) including residues 786–992, which encompass the extracellular domain of the protein (residues 22–958). Based on the RNA sequencing analysis in the proband and in silico prediction of the translated aberrant transcript, it is evident that a significant proportion of the extracellular topological domain of the protein is likely to be lost including the bulk of the N‐terminal region (Figure 1g). It is the first report where aberrant splicing in *EMC1* is shown to affect the mRNA and potentially lead to the disease phenotype.

A rare homozygous missense variant (c.430G>A; p.Ala144Thr) in *EMC1* has been implicated in a family with nonsyndromic retinitis pigmentosa based on the functional role of the gene in rhodopsin biosynthesis in *Drosophila* (Abu‐Safieh et al., 2013). Our findings are in line with the only other report of *EMC1* mutations in individuals affected with developmental delay, hypotonia, scoliosis, and cerebral atrophy. Mutations have been reported in four families of which two had homozygous missense variants (p.Thr82Met; p.Gly868Arg), one had a homozygous frameshift mutation (Pro874Argfs*21), and the fourth family had a nonsynonymous *de novo* heterozygous missense variant (p.Gly471Arg). The individuals had varying severity of global developmental delay, speech delay, microcephaly, and abnormal ophthalmological features. Brain imaging was suggestive of cerebellar atrophy and foreshortened corpus callosum in all four families. Some of the patients had dystonia and increased tone in the extremities. They also displayed mild dysmorphic features of deep‐set eyes, short philtrum, retrognathia/micrognathia, and gingival hyperplasia (Harel et al., 2016). The severity of the phenotype reported was independent of the type of mutation or its location on the protein. In addition to the phenotypes described in literature (Table S1), our patient displayed scaphocephaly, recurrent seizures, brain stem atrophy, and severe muscle wasting. The severity of the phenotype observed is likely due to the loss of more than half of the intact functional protein. The phenotype variability may also be due to the type of mutation or its effect on the functioning of the EMC complex (protein folding, ER‐mitochondria tethering, and assembly of multi‐pass membrane proteins). However, additional functional studies are needed to delineate the exact mechanism underlying the disease phenotype.

In summary, we have identified a novel homozygous splice variant in *EMC1* in a child with global developmental delay. Based on the limited clinical case reports, there seems to be varying disease severity and progression, in spite of the patients sharing recognizable features of developmental delay, scoliosis, hypotonia, and cerebellar atrophy. Additional functional studies are required for appreciating the phenotype–genotype correlations and underlying disease mechanism. We would also like to emphasize the importance of screening intronic splice mutations in routine genetic diagnosis. This may further our understanding of indistinct clinical phenotypes and their relevance to these disease conditions.

## CONFLICT OF INTEREST

None.
