# Supplementary material for: A novel splice variant in EMC1 is associated with cerebellar atrophy, visual impairment, psychomotor retardation with epilepsy
Source: Mol Genet Genomic Med. 2017 Dec 22;6(2):282–7. doi: 10.1002/mgg3.352 (PMC5902391; doi:10.1002/mgg3.352)
Supplement: Supplementary file 1 [file MGG3-6-282-s001.docx]

**Supplementary table 1: Comparison of clinical features observed of this study with those reported in literature.**

| **Features** | **This study** | **Phenotype from published cases (Harel *et al*., 2016);** |
| --- | --- | --- |
| **Dysmorphism:**  Scaphocephaly  Deep set eyes | +  + | - (none)  + (2/4) |
| Global developmental delay  Seizures | +  + (recurrent seizures with onset at 5month of age) | + (4/4)  1 pt had subclinical seizures |
| Microcephaly | + | + (3/4) |
| Truncal hypotonia  Reduced/absent DTR | +  + | + (4/4)  + (3/4) |
| Limb dystonia  Muscle wasting | +  + | + (2/4)  - (none) |
| Scoliosis | + | + (3/4) |
| **Opthalmic features:**  Cortical visual impairment  Optic disc pallor  Abnormal VEP, ERG  Myopia | +  +  Not done  - | + (4/4)  +  + (4/4)  + (1/4) |
| **MRI Features:**  Cerebellar atrophy with cortical atrophy  Brain stem atrophy  Thin corpus callosum | +  +  - | + (4/4)  - (none)  + (4/4) |
